# Polymer Gels Based on PAMAM Dendrimers Functionalized with Caffeic Acid for Wound-Healing Applications

**DOI:** 10.3390/gels11010036

**Published:** 2025-01-04

**Authors:** Ricardo I. Castro, Wendy Donoso, Franko Restovic, Oscar Forero-Doria, Luis Guzman

**Affiliations:** 1 Multidisciplinary Agroindustry Research Laboratory, Instituto de Ciencias Aplicadas, Carrera de Ingeniería en Construcción, Universidad Autónoma de Chile, Talca 3467987, Chile; 2 Departamento de Bioquímica Clínica e Inmunohematología, Facultad de Ciencias de la Salud, Universidad de Talca, Talca 3460000, Chile; wdonoso@utalca.cl; 3 Centro de Biotecnología de Sistemas, Facultad de Ciencias de la Vida, Universidad Andrés Bello, Santiago 8370186, Chile; franco.restovic@unab.cl; 4 Departamento de Ciencias Básicas, Facultad de Ciencias, Universidad Santo Tomás, Talca 3460000, Chile; oforero@santotomas.cl

**Keywords:** gel-PAMAM dendrimer, wound healing, caffeic and cinnamic acid, synthesis polymers gels, assay in vitro

## Abstract

The wound-healing process has usually been related to therapeutic agents with antioxidant properties. Among them, caffeic acid, a cinnamic acid derivative, stands out. However, the use of this natural product is affected by its bioavailability and half-life. Nowadays, different approaches are being taken to improve the above-mentioned characteristics, as many active surface groups are present in polyamidoamine (PAMAM) dendrimers; without the need for extra cross-linking agents, physical gels are created by interactions such as hydrogen bonds, van der Waals forces, or π–π interactions based on the modification of the surface. One of these is functionalization with dendrimers, such as the poly(amidoamine) (PAMAM) family. To evaluate the effectiveness of functionalizing caffeic acid with PAMAM dendrimers, the in vitro and in vivo wound-healing properties of gel-PAMAM G3 conjugated with caffeic acid (GPG3Ca) and its precursor, cinnamic acid (GPG3Cin), were studied. The results showed no cytotoxicity and wound-healing activity at a concentration of 20 μg/mL in HaCaT cells with the GPG3Ca. Additionally, the ability to activate molecular mediators of the healing process was evidenced. Furthermore, GPG3Ca potentiated the in vivo wound-healing process. The positive effects and lack of cytotoxicity at the used concentration of the synthesized GPG3Ca on the wound-healing process could position it as an effective agent for wound-healing treatment.

## 1. Introduction

Wound healing is an important process because delayed wound healing could result in skin diseases such as severe edema and chronic ulcers [1]. An ideal process should take a short time to develop without any side effects for a successful closure [2].

Wound healing is a multifaceted process consisting of sequential yet overlapping phases, including hemostasis/inflammation, proliferation, and remodeling phases [3]. Several reviews of the current knowledge about the cellular and molecular pathways are provided in the literature [4,5,6]. Under normal physiological conditions, the process is highly efficient; however, when the tissue fails to regain structural and functional integrity, it results in chronic wounds, particularly in the elderly population, with progressively prevalent disease conditions such as vascular diseases, diabetes, and obesity [7].

The wound-healing process has been associated with the antioxidant properties of therapeutic agents, as these accelerate the wound-healing process by increasing the synthesis of colloids and removing radicals such as free oxygen radicals [8,9]. There are several studies regarding the therapeutic benefits of plants or substances obtained from plants, such as flavonoids or phenolic compounds, on wound healing [10,11]. Among the main compounds present in plants and plant extracts is caffeic acid (3,4-dihydroxycinnamic), which is a cinnamic acid derivative, also called phenylpropanoids. This polyphenol is widely distributed in many food sources and can be found in traditional medications [12,13,14].

Molecules such as flavonoids and polyphenols have been reported as important cell proliferation stimulating agents in addition to promoting the regeneration of the damaged epithelium and accelerating the healing of the wound [12,15,16]. However, it is known that the stability of many of these molecules can vary depending on the conditions in which they are applied. It has been demonstrated that polymeric systems such as dendrimers, hydrogels, micelles, and gels improve the stability of bioactive compounds under varying conditions. In addition to increasing solubility, these systems shield delicate substances against deterioration brought on by changes in pH, light, and temperature [17,18].

In general, they are slightly soluble in alcohols but insoluble in oils and apolar solvents, meaning that their bioavailability and half-life are strongly affected by how they are delivered [19,20]. Nowadays, the physicochemical characteristics of different compounds can be modified by functionalization with other macromolecules.

Polymer gels are highly versatile materials with applications in memory switches, sensors, controlled drug release, artificial muscles, and enzyme embedding. They are classified into chemical and physical gels based on their crosslinking type [21]. While chemical gels often rely on additives like crosslinking agents to enhance performance, particularly in biomedical applications, physical gels are formed through hydrogen bonding and other non-covalent interactions, van der Waals forces, or π–π interactions based on the modification of the surface, generating alternatives of polymer gels based on functionalized PAMAM dendrimers [22].

Among these, we can highlight the poly amidoamine (PAMAM) family, constituted by an ethylenediamine core, repetitive branches of tertiary amines and polyamides, plus a terminal layer of primary amines [23,24]. Due to their versatile surface chemistry, PAMAM dendrimers allow the covalent attachment of different functional groups, modulating their physicochemical properties for different nanomedical purposes [25,26,27]. Moreover, under physiological conditions, the surface modification of the PAMAM dendrimers with different compounds mitigates the nonspecific cytotoxicity and hemolysis evidenced mainly in the fourth PAMAM dendrimer generation (or higher) due to their polycationic structure [28,29].

As a result of the importance of natural compounds such as caffeic acid in the wound-healing process and the ability of PAMAM dendrimers to improve the physicochemical characteristics of molecules attached to their surface, the aim of this research is to study the in vitro and in vivo wound-healing properties of PAMAM G3 conjugated with caffeic acid and its precursor, cinnamic acid.

## 2. Results and Discussion

### 2.1. Characterization of GPG3Ca and GPG3Cin

**Figure 1:**
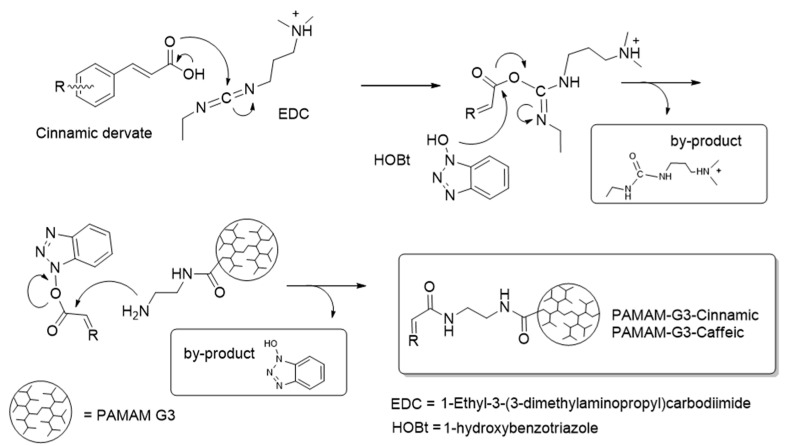
Synthesis of GPG3Ca and GPG3Cin in the reaction formation of amides.

In the preparation of amides, the coupling reagent 1-Ethyl-3-(3-dimethylaminopropyl) carbodiimide (EDC) is commonly used in addition to 1-hydroxybenzotriazole (HOBt) as a catalyst in a ratio of 10:1 [30,31,32]. In this reaction, EDC reacts with carboxylic acid in order to form an active O-acylisourea intermediate, with all of this catalyzed by HOBt. The latter activates the carbon of carboxylic acid, followed by the remotion of the urea byproduct by aqueous extraction. Afterwards, the HOBt is displaced by nucleophilic attack from primary amino groups of the amide (Figure 1).

**Figure 2:**
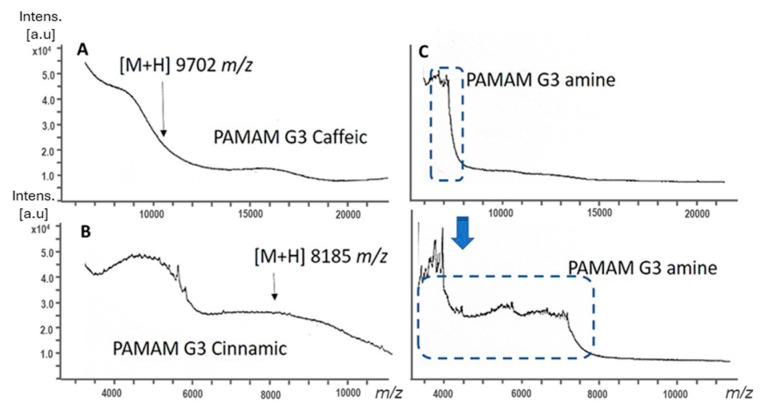
MALDI-TOF spectra of (**A**) GPG3Ca; (**B**) GPG3Cin; (**C**) PAMAM G3.

The mass spectra in Figure 2 provide crucial insights into the composition and molecular interactions of the PAMAM G3 dendrimer. Signals at 9702 and 8185 *m*/*z*, corresponding to GPG3Ca and GPG3Cin (Figure 2A,B), respectively, indicate the successful conjugation of these compounds to the dendrimer. These peaks demonstrate a significant increase in molecular weight compared to the unmodified PAMAM G3 dendrimer, which has an approximate mass of 7300 Da. This increase in *m*/*z* values provides direct evidence of the covalent attachment of GPG3Ca and GPG3Cin to the dendrimer core from the organic synthesis proposed in this study.

Furthermore, Figure 2C reveals the presence of both singly charged molecular ions and doubly charged molecular ions, with *m*/*z* values corresponding to the PAMAM G3 dendrimer. The singly charged ion aligns with the expected molecular weight of the dendrimer PAMAM G3, while the doubly charged ion confirms the intactness and ionization efficiency of the dendrimer during MALDI-TOF analysis.

The estimated *m*/*z* values allowed to determine the approximated number of molecules conjugated per mol of PAMAM G3: 15 molecules of caffeic acid and 18 molecules of cinnamic acid per mol for GPG3Ca and GPG3Cin.

**Figure 3:**
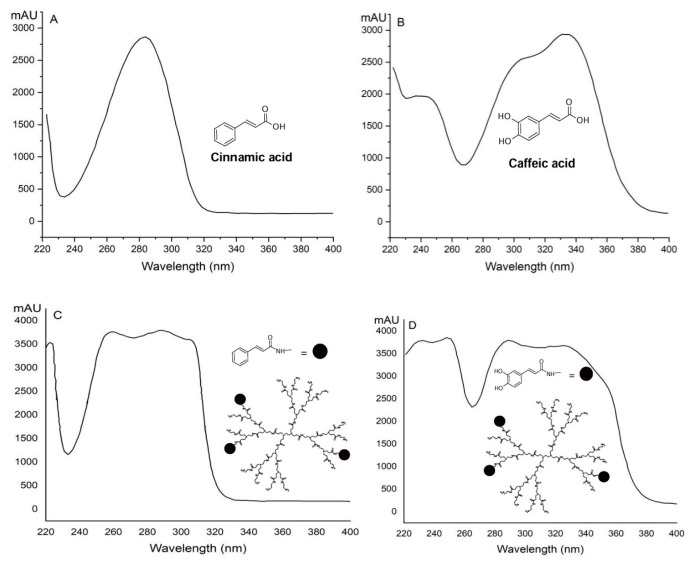
UV–visible spectra of (**A**) cinnamic acid; (**B**), caffeic acid; (**C**), GPG3Cin; (**D**) GPG3Ca.

Additionally, the conjugation of GPG3Ca and GPG3Cin was confirmed by UV–visible analysis (Figure 3).

**Figure 4:**
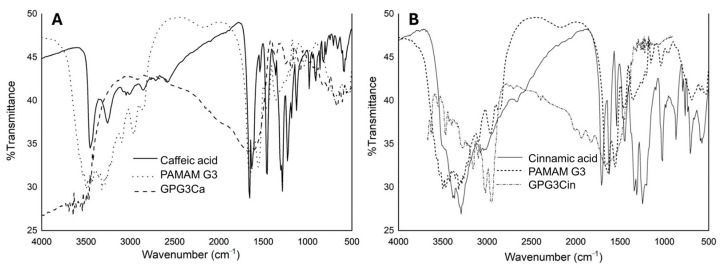
FTIR spectra of (**A**) caffeic acid: solid line, PAMAN G3: dotted line, and GPG3Ca: dashed line; (**B**) cinnamic acid: solid line, PAMAN G3: dotted line, and GPG3Cin: dashed line.

A vibration band at 3500 cm^−1^ in the FT-IR spectra of the caffeic and cinnamic acids (Figure 4A,B) indicates the stretching of the O-H bond properties of the carboxylic functional group. Additionally, both hydroxycinnamic acids exhibit a band at 1690 to 1652 cm^−1^, which corresponds to the stretching vibration of the C=O carbonyl group.

The aromatic ring’s C-C/C-H stretching and bending vibrations are characterized by the presence of bands between 1609 and 1386 cm^−1^, whereas the phenol alcohol’s O-H and C-H bending vibrations produce the distinctive peaks in the region between 1314 and 1180 cm^−1^. The caffeic acid has the latter peaks (Figure 4A). After the amidation conjugation reaction between caffeic and cinnamic acids and PAMAN G3, some signals disappear, and others are shifted to lower wavenumber values. In the FT-IR spectra of the conjugated GPG3Ca and GPG3Cin (Figure 4A,B), a band is observed at 1643 cm^−1^, which corresponds to the C=O stretching vibration of amide I, and the N-H bonding vibration of amide II at 1531 cm^−1^ is shifted by 9–12 cm^−1^ in the resulting conjugated amide.

The conjugated GPG3Ca also showed a signal stretching between 3450 and 3300 cm^−1^ that corresponds to the hydroxyl groups of the caffeic acid (Figure 4A). Another signal that confirmed the conjugation of the PAMAM G3 dendrimer is the decrease in the intensity of the peak at 3200 cm^−1^, which can be assigned to the N-H bending stretching vibration (NH_2_ of the periphery) of the PAMAM G3 dendrimer and the formation of new intense peaks at 1640 and 1630 cm^−1^, which correspond to the N-H bending and C-N stretching of the amide formed between the acid and the PAMAM G3 dendrimer [33]. The strongest stretching band located at 1688 cm^−1^ in caffeic acid spectrum is absent in the conjugate GPG3Ca. This corresponds to the stretching vibration of the carboxylic acid, which indicates that the conjugation to the PAMAN G3 was through this functional group.

**Figure 5:**
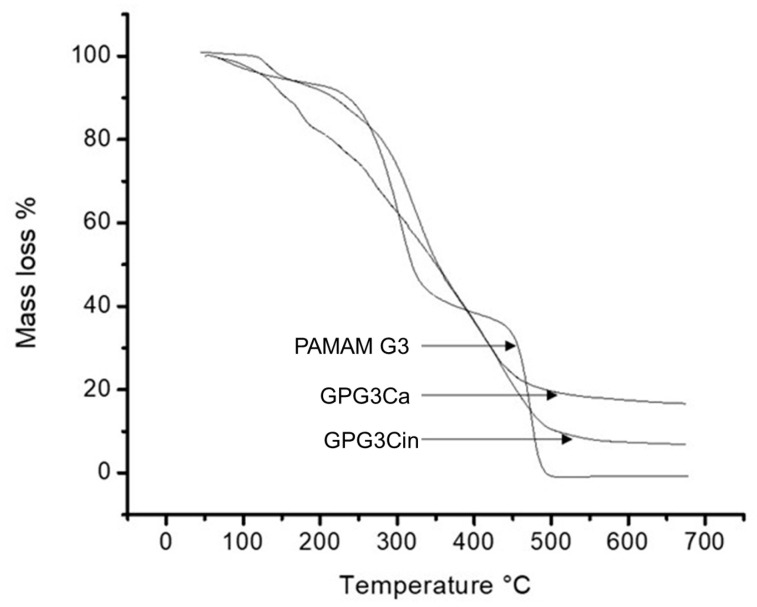
Thermograms of GPG3Ca, GPG3Cin, and PAMAM G3 at temperatures between 50 and 700 °C.

The thermogravimetric analysis of the conjugated dendrimers showed an initial degradation temperature lower than the PAMAM-G3 (Figure 5) due mainly to the decomposition or the volatilization of water [34]. At temperatures above 500 °C, the conjugated dendrimers exhibit more thermic stability, possibly due to the conjugation with caffeic and cinnamic acids, ascribed to changes in intramolecular and intermolecular interactions brought on by the chemicals that are bound. These changes may compromise the dendrimer’s natural structural integrity, which is more susceptible to thermal decomposition, increasing its thermal breakdown in the stages of heating associated with the decrease in crystalline state [35].

**Figure 6:**
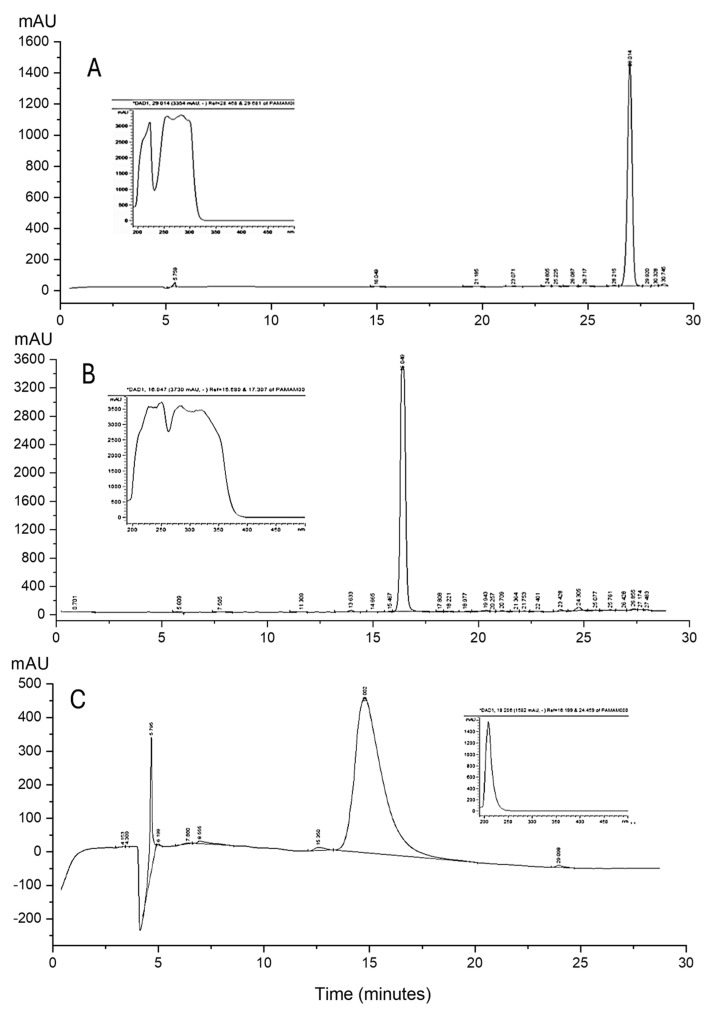
HPLC chromatogram of (**A**) GPG3Ca; (**B**) GPG3Cin; (**C**) PAMAM G3.

For its part, the HPLC analysis was carried out to evidence the polydispersity of the PAMAM G3 and its derivatives (Figure 6). Before the analysis, the conjugated dendrimers were purified by a dialysis membrane, and the chromatograms did not show small peaks or band broadening of the main peak that could be attributed to structural defects present during the synthesis of the PAMAM derivatives. The retention times (rt) were 16.04 min for GPG3Ca and 29.01 min for GPG3Cin, different from PAMAM G3 amine (18.00 min).

The polydispersity of the samples (conjugated dendrimers) was estimated qualitatively by assessing the peak width at half height (Wh) compared with PAMAM G3 amine (Table 1).

Poly(amidoamine) (PAMAM) dendrimers are a class of polymers characterized by a low polydispersity and dendritic structure. Additionally, the possibility of the conjugation of their surface groups has contributed to make PAMAM dendrimers suitable for biological applications [36,37,38]. Although PAMAM dendrimers have low polydispersity, generational defects (oligomers and trailing generations) have been reported. Within the latter, branching defects such as intramolecular loops and missing arms have been evidenced. These defects could lead to dendrimers with significantly higher and lower molecular weights and diameters [39,40]. These dendrimers can be evidenced as small peaks and band broadening of the main peak, as shown for the commercial PAMAM G3 amine used in this study, a situation that was not observed in the synthesized GPG3Ca and GPG3Cin.

### 2.2. Cell Viability

**Figure 7:**
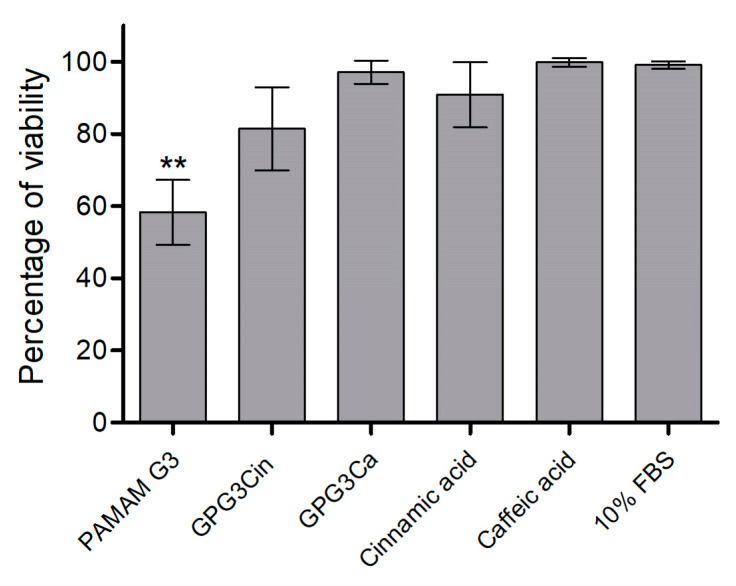
HaCaT Cell viability at a concentration of 20 μg/mL of the different molecules studied. ** *p* < 0.01.

The PAMAM G3 dendrimer and the derivatives GPG3Ca and GPG3Cin did not show a decrease in the viability of HaCaT cells at 2, 5, and 10 μg/mL (Table 2). However, 20 μg/mL of the PAMAM G3 dendrimer showed a statistically significative decrease (*p* < 0.01) in the viability of the HaCaT cells (Figure 7).

Kitchens, K.M. (2017) showed that Caco-2 Cells treated with different concentrations and generations of PAMAM-NH_2_ presented a membrane disruption and loss of microvilli. Also, the use of PAMAM G4-NH_2_ at a concentration of 0.1 mM or higher induced membrane disruption, whereas the use of PAMAM G3.5 (COOH) dendrimer at the same concentration did not induce any membrane damage. Additionally, it was evidenced that higher generations of cationic PAMAM dendrimers showed increased membrane damage at the same concentration [41]; thus, is thought that the cytotoxicity is the result of the interaction between positively charged dendrimers and negatively charged cell surfaces [42,43,44]. Moreover, it was proven that PAMAM NH_2_ dendrimers possess a cytotoxicity profile depending on the surface charge, generation, and concentration, suggesting that a modification of its periphery could diminish its cytotoxicity [45]. The cytotoxicity of cationic dendrimers and the conjugation of its surface to diminish the cytotoxicity was reported in PAMAM molecules both in vivo and in vitro [46]. It was shown in HEK cells that PAMAM G4 dendrimers induced 50% of cell viability at a concentration of 50 μM after 24 h of incubation and decreased below 25% when a concentration of 500 μM was used. On the other hand, PAMAM with polyethylene glycol (PEG) and folic acid (FA) were non-cytotoxic at the entire range of concentration used (1–500 μM); a similar effect was found in the present study, where PAMAM G3 at a concentration of 20 μg/mL induced a cell viability below 60%, but the conjugated GPG3Ca and GPG3Cin showed no cytotoxicity at the entire studied range. In general, neutral or anionic dendrimers show very low toxicity [47]; however, cationic dendrimers such as PAMAM dendrimers show an increased toxicity as the generation of the dendrimer increases [48]. The cytotoxicity mechanism includes membrane cell destabilization and oxidative stress, which induce an increase in the reactive oxygen species, which could lead to apoptosis [49].

### 2.3. In Vitro Scratch Assay

**Figure 8:**
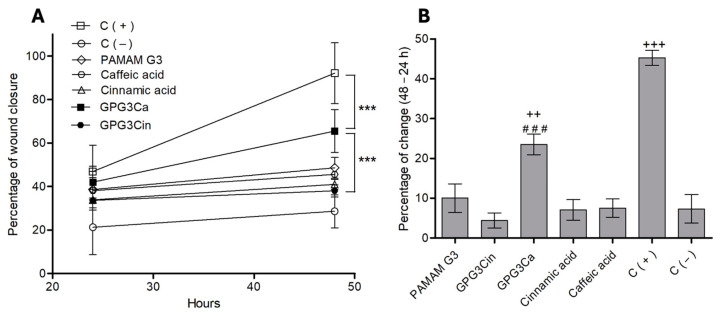
In vitro scratch assay. (**A**) Wound-closure percentages at 24 and 48 h. (**B**) Percentage of change between 24 and 48 h. *** *p* < 0.001 compared to GPG3Cin and the positive control; ++ *p* < 0.01 compared to the positive control; ### *p* < 0.001 compared to PAMAM G3, GPG3Cin, cinnamic and caffeic acid, and negative control; +++ *p* < 0.001 compared to the rest of the molecules.

The scratch assay showed that at 24 h of incubation, no statistically significant differences were found between the PAMAM derivatives and controls, but at 48 h of incubation, the positive control (10% FBS) showed nearly 96 ± 5% wound closure; meanwhile, GPG3Ca showed a better percentage of wound closure compared with GPG3Cin (*p* < 0.001), but it was less effective (*p* < 0.001) than the positive control (Figure 8A). In turn, the percentage of change between 24 and 48 h of GPG3Ca was 23.5 ± 2.6%, which was lower (*p* < 0.01) than the positive control (45.3 ± 1.9%) but better (*p* < 0.001) compared to GPG3Cin and the negative control (Figure 8B).

Scratch assays commonly reflect the migration and proliferation process of the cell. For that, cells are cultivated in a dish to form a confluent monolayer, and an artificial wound is made by a mechanical scratch and images taken (over 12–24 h) of the resulting collective cell spreading, driven by combined cell migration and proliferation [50,51].

Usually, the wound-healing properties of pure compounds or plant extracts are studied by the scratch assay with concentrations that can range from μg/mL to mg/mL. Merfort et al. (2009) studied the wound-healing property by scratch assay (3T3 fibroblast) of hexane and ethanolic extracts from *Calendula officinalis*, using concentrations that ranged from 1 to 10 μg/mL of the crude extract and from 10 to 50 μg/mL of the isolated compounds. The results showed that the ethanolic extract increased cell numbers to 60.80% ± 4.36 and 70.53% ± 2.64 at concentrations of 1 and 10 μg/mL, respectively. Nevertheless, when the isolated compounds obtained from the Calendula were tested at 10 and 50 μg/mL, they showed an increase in cell number of 37.87% ± 2.32 and 73.30% ± 2.43, respectively [52]. Synthesized compounds such as chitosan and its derivatives have also been tested for their wound-healing property. Felice et al. (2015) tested high- and low-molecular-weight chitosan derivatives at a concentration of 10 μg/mL in DMEM with 1% FBS for 24 h at 37 °C, showing that high-molecular-weight quaternary ammonium–chitosan conjugates were more effective in promoting cell migration than the non-thiolated conjugates and the chitosan chlorhydrate [53].

Many reports confirm that the wound-healing activity of different natural products or synthetic compounds work with optimal concentrations over 10 μg/mL, with some products or compounds even with optimal concentrations in the range of mg/mL [54,55,56]. Here, we report that an optimal concentration of 2 μg/mL can exceed a 60% wound closure rate after 48 h of incubation, a concentration that is significantly lower than most of those reported but greater than the physiological concentrations of most of the factors involved in the healing process, such as transforming growth factor-b family (TGF-β), interleukin (IL), and angiogenesis factors (i.e., vascular epidermal growth factor).

### 2.4. Gene Expression Analysis Using Real-Time PCR

**Figure 9:**
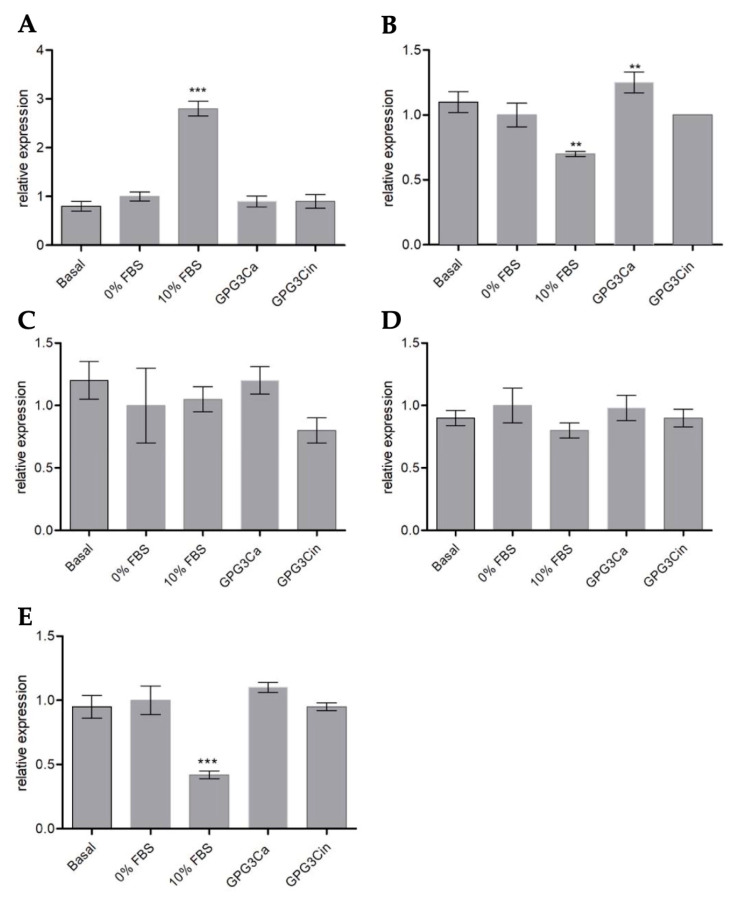
Relative expression of (**A**) Ki67; (**B**) Cyclin D1; (**C**) IL-8; (**D**) MMP 1; (**E**) KRT 17. ** *p* < 0.01 and *** *p* < 0.001 compared to the basal expression.

For the analysis of gene expression, the transcriptional levels of genes that are key for the activation and regulation of the wound-healing process were evaluated (Figure 9A–E), such as genes involved in keratinocytes proliferation (Ki-67, Cyclin D1) and activation (KRT17), epidermal growth markers (IL-8), and migration markers and extracellular matrix productions-related genes (MMP1).

No statistically significant differences were found between the negative control (0% SFB) and the different gene expression markers (Ki-67, KRT17, IL-8, and MMP1). However, when comparing Cyclin D1 gene expression and the negative control, there was a significant increase (*p* < 0.01) in the expression of this gene (Figure 9B), whose product is a protein belonging to the family of cyclins characterized by drastically increased cell cycle.

It has been previously reported that various target genes in the NF-kB signaling pathway are linked to cell proliferation and wound healing, one of those being Cyclin D1, which is associated with cell proliferation and wound healing [57,58]. Cyclin D1 regulates proliferation, connecting the extracellular signaling environment to cell cycle progression. The expression of cyclin D1 is very responsive to proliferative signals, including Ras growth factor receptors and its downstream effectors. Additionally, its expression increases upon stimulation of quiescent cells to enter the cell cycle [57,59,60]. Thus, the significative increment induced by GPG3Ca is important evidence of the direct effect of this compound over the stimulation of the wound-healing process.

### 2.5. In Vivo Wound-Healing Activity

**Figure 10:**
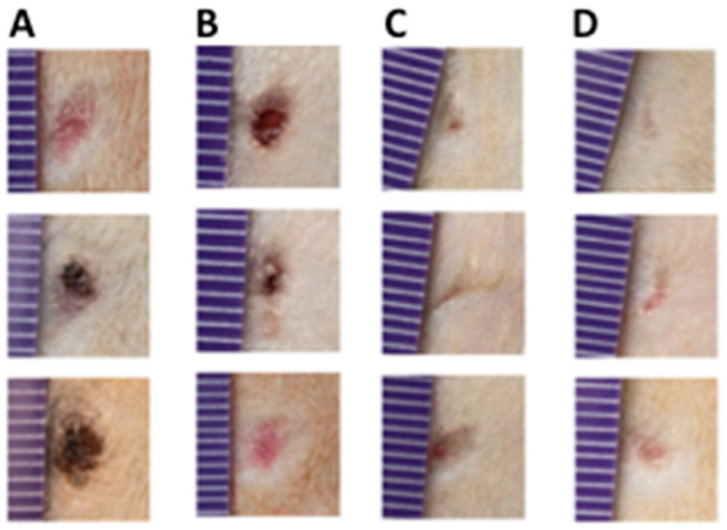
Representative images of the in vivo wound-healing process (day 9). The columns represent (**A**) saline control group; (**B**) base cream group; (**C**) GPG3Ca 0.05% group; (**D**) GPG3Ca 0.1% group.

At first, the in vivo wound-healing activity protocol duration was expected to reach 16 days; however, all animals were sacrificed on day 9 due to the fast closing of the wounds in those creams supplemented with the GPG3Ca, both at 0.05% and 0.1%, compared to both controls (Figure 10).

**Figure 11:**
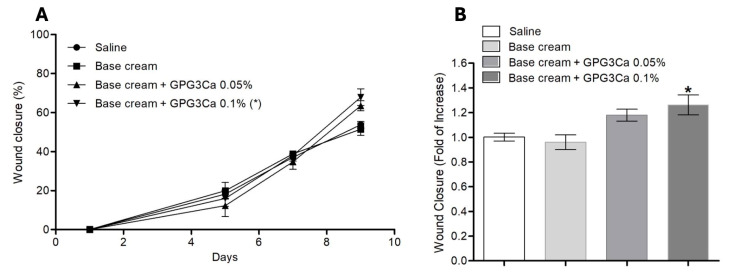
(**A**) Wound-healing process (9 days) of the different groups studied and (**B**) increase (folds) in wound closure at the end of the assay. * *p* < 0.05 compared to saline and base cream controls.

In addition, the base cream did not show an increase with respect to the saline (Figure 11A,B); however, the base cream supplemented with GPG3Ca (0.05 and 0.1%) showed an increase in the percentage of healing, showing a statistically significant difference (*p* < 0.05) between the GPG3Ca 0.1% and the respective controls (saline and base cream) (Figure 11B).

At the same time, the statistical analysis of the concentration of lactate dehydrogenase (LDH) of each of the groups did not show a significant difference among the different experimental groups, presenting a mean of determinations of 98 ± 33 U/L.

**Figure 12:**
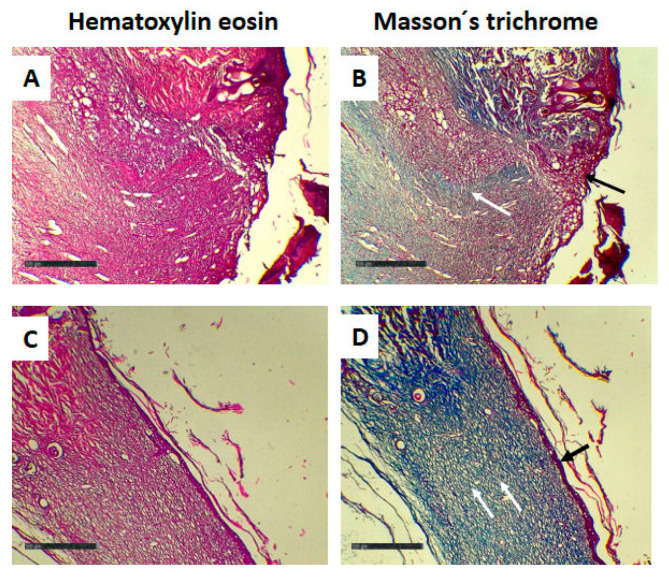
Histopathological analysis at day 9 of base cream group (**A**,**B**) and GPG3Ca 0.1% group (**C**,**D**). In (**A**,**B**), a partially recovered epidermis was observed (black arrow, image (**B**)); also, granulation tissue, neovascularization, and thin collagen fibers in a blue tone with Masson trichrome staining were observed in the dermis (white arrow, image (**B**)). In image (**C**,**D**), a completely re-epithelialized epidermis was observed (black arrow, image (**D**)); in addition, in the dermis, thicker collagen fibers with Masson trichrome staining and little granulation tissue were observed (white arrow, image (**D**)). Magnification (40×), scale bar 500 µm.

On the other hand, the histopathological results showed that all samples presented a healing stage with proliferation of fibroblasts and collagen production concomitant with the re-epithelialization of the injury area but with no differences among the different groups studied (Figure 12).

The histopathological analysis did not reveal differences between the different treatments. However, the difference between the latter and the macroscopical results (in vivo wound closure) could be attributed to the subjective scoring system (none, mild, moderate, or severe) used in the histopathological analysis. An immunohistochemical analysis could lead to a better characterization of the wound stage of every group, avoiding bias in the interpretation.

## 3. Conclusions

This study successfully synthesized and characterized novel GPG3Ca and GPG3Cin, demonstrating their non-toxicity at concentrations up to 20 μg/mL. GPG3Ca exhibited significant wound-healing activity in vitro at a concentration of 2 μg/mL, as evidenced by enhanced cell migration, proliferation, and gene expression. These in vitro findings were corroborated by in vivo studies, where the dendrimer facilitated faster wound closure and improved histopathological outcomes, including re-epithelialization and collagen production, at a concentration of 0.1% (GPG3Ca). While the histopathological analysis did not show significant differences among the groups, the overall wound-healing efficacy of GPG3Ca was evident from the macroscopic results. This discrepancy highlights the need for more refined histopathological evaluation methods, such as immunohistochemical analysis, to better characterize the wound-healing stages. The results of this study position GPG3Ca as a promising candidate for wound-healing applications, warranting further investigation and development for clinical use.

## 4. Materials and Methods

### 4.1. Materials

PAMAM G3 (Dendritech, Midland, MI, USA), cinnamic acid (Sigma-Aldrich, St. Louis, MO, USA), caffeic acid (Sigma-Aldrich, St. Louis, MO, USA), dimethyl sulfoxide (DMSO; Sigma-Aldrich), 1-[3-(dimethylamino)propyl]-3-ethylcarbodiimide hydrochloride (EDC; Sigma-Aldrich), Hi-droxybenzotriazole (HOBt; Sigma-Aldrich), membrane (cut-off 4000 Da) (Spectrum Laboratories, Rancho Dominguez, CA, USA), 2,5-dihydroxybenzoic acid (Sigma-Aldrich), trifluoroacetic acid (TFA) (Sigma Aldrich), 2,3-bis(2-methoxy-4-nitro-5-sulfophenyl)-2-h-tetrazolium-5-carboxanilide (Fisher scientific, Waltham, USA), and mili-Q water 18 mΩ were utilized.

### 4.2. Methods

#### 4.2.1. Functionalization of PAMAM G3 Dendrimer with Cinnamic Acid (GPG3Cin) and Caffeic Acid (GPG3Ca)

The functionalization of PAMAM G3 with cinnamic acid and caffeic acid was carried out in a glass flask by condensation between the carboxyl group of cinnamic and caffeic acid and PAMAM G3 dendrimers. For this, 1.0 mmol of cinnamic and caffeic acid was dissolved in anhydrous dimethyl sulfoxide, and then, 1.0 mmol of 1-[3-(dimethylamino)propyl]-3-ethylcarbodiimide hydrochloride (EDC) and 0.1 mmol of Hidroxybenzotriazole (HOBt) were added, followed by 0.05 mmol of PAMAM G3 dendrimer under a nitrogen atmosphere (Figure 1); afterwards, the reaction mixture was stirred for 72 h at room temperature. The reaction product was dialyzed with a membrane (cut-off 4000 Da), and the conjugated PAMAM dendrimer obtained was lyophilized (Freezone 6, LabConco, Kansas City, MO, USA) [33].

#### 4.2.2. Characterization of GPG3Ca and GPG3Cin

##### MALDI-TOF Analysis

The molecular weights of GPG3Ca and GPG3Cin were determined with a MALDI-TOF spectrometer (Bruker Daltonics Flex Control, Bremen, Germany) with a pulsed nitrogen laser (λ = 337 nm), operating in positive ion reflector mode, with 20 KV acceleration voltage with a matrix of 2,5-dihydroxybenzoic acid.

##### FT-IR Spectroscopy

The infrared spectra of the conjugated PAMAM G3 dendrimers were studied by FT-IR in a Nicolet Nexus 470 spectrometer with KBr film cells with a spectral range of 3800 to 600 cm^−1^ with an average of 32 scans and a resolution of 4 cm^−1^.

##### Thermogravimetric Analysis (TGA)

The thermogravimetric analyses of GPG3Ca and GPG3Cin were performed in an TGA-Q500 analyzer (TA-instruments, New Castle, DE). The samples were heated at 20 °C min^−1^ between 50 °C and 700 °C, using air as a reactive gas, with a flow of 60 mL min^−1^. Additionally, 40 mL min^−1^ of N_2_ was used as protection gas for the electronic balance. For each analysis, around 5 mg of dendrimers were used in a Pt crucible.

##### Reversed-Phase High-Performance Liquid (HPLC) Analysis

To determine the polydispersity of the synthesized PAMAM derivatives, an HPLC analysis was carried out using an HPLC 1260 Infinity Model (Agilent ChemStation, 1200, Santa Clara, CA, USA) with a photodiode array detector (Agilent ChemStation, 1200, USA). The separation of the analytes was performed with a LiChrospherRP-18 HPLC column of 250 mm (5 μm) with guard columns. Briefly, each conjugated PAMAM G3 dendrimer was weighed (1.0 mg), dissolved in 5 mL of water/trifluoroacetic acid (TFA) 0.14% (*v*/*v*), and membrane-filtered (0.45 μm pore size) with a flow rate of 0.5 mL/min. The mobile phase for elution of solution A was TFA 0.14% (*v*/*v*) in water and for solution B was acetonitrile, with an initial condition of 90% of solution A and 10% of solution B, until it reached 50% of solution A, within an interval of 30 min. The injection volume in each sample was 25 μL and was determined by a direct readout of the sample absorbance at 280 nm.

##### UV–Vis Spectroscopy

The conjugations of caffeic and cinnamic acids were monitored by a UV–vis absorption spectrophotometer at room temperature in a range from 190 to 500 nm, using a 1 cm long quartz cuvette, in water/TFA 0.14% (*v*/*v*).

#### 4.2.3. Determination of Cell Viability

Cell viability was carried out by incubating GPG3Ca and GPG3Cin at concentrations of 2, 5, 10, and 20 μg/mL with immortalized human keratinocytes HaCaT cells for 48 h (100% confluence) in Dulbecco’s modified Eagle’s medium (DMEM) containing 10% fetal bovine serum (FBS), penicillin (100 U/mL), and streptomycin (100 μg/mL) at 37 °C in a humidified incubator containing 5% CO_2_. Finally, the cells were incubated with the reagent sodium (2,3-bis(2-methoxy-4-nitro-5-sulfophenyl)-2-h-tetrazolium-5-carboxanilide), also known as XTT, in which the salts of this reagent are reduced by the mitochondrial dehydrogenases of the viable cells to formazan crystals, indicating the number of living cells present in the cultures. As controls, phosphate-buffered saline (PBS) and cinnamic and caffeic acids were used at equimolar concentrations according to the degree of conjugation of each molecule.

#### 4.2.4. In Vitro Scratch Assay

HaCaT cells were plated until reaching the complete coverage of the well surface. The scratch assay was performed by scratching the cell culture with a 200 μL pipette tip to form the wound. Cell debris was later eliminated by removing the culture medium and washing the cells with PBS–Dulbecco (DPBS). Then, culture medium containing the conjugated dendrimers (2 μg/mL) was added. As a negative control, culture medium without fetal bovine serum (0% FBS) was used, and 10% of FBS was added as a positive control. Images of the wound area were taken at 0, 24, and 48 h, using a camera connected to an inverted microscope. The migration radius of the cells was calculated using the Image-J software (1.51d) to analyze the images by the distance traveled by the cells, comparing them with the negative control.

#### 4.2.5. Gene Expression Analysis Using Real-Time PCR

HaCaT cells were incubated for 48 h with both dendrimers at a concentration of 2 μg/mL. Cells were scraped and collected, and RNA was extracted using the Direct-zol RNA Miniprep kit (Zymo Research, Irvine, UAS), following the manufacturer’s instructions. The quantification of the RNA was carried out by measuring the absorbance in a Tecan Infinite M200 Pro spectrophotometer.

For the analysis of gene expression, the transcriptional levels of genes that are key for the activation and regulation of the wound-healing process were evaluated, such as genes involved in the proliferation of keratinocytes like Ki-67 (forward primer CTTTGGTGGGCACCTAAGACC; reverse primer TGATGGTTGAGGTCGTTCCTTG) and Cyclin D1 (forward primer GAACAAACAGATCATCCGCAAAC; reverse primer GCGGTAGTAGGACAGGAAGTTG); genes involved the activation of keratinocytes, such as keratins KRT17 (forward primer AGGTGCGTACCATTGTGGAA; reverse primer ATCAGGCAAGGAAGCATGGG); epidermal growth markers, such as IL-8 (forward primer ATGACTTCCAAGCTGGCCGT; reverse primer TCTCAGCCCTCTTCAAAAACTTCT); and markers of migration and production of extracellular matrix, such as MMP1 (forward primer TGTGGACCATGCCATTGAGA; reverse primer TCTGCTTGACCCTCAGAGACC). GADPH was used as a reference gene expression (forward primer TGCACCACCAACTGCTTAGC; reverse primer GGCATGGACTGTGGTCATGAG). The gene expression was performed in a Real-Time PCR System Step One Plus (Applied Biosystems, Carlsbad, USA); two microliters of purified RNA were used as a template in RT-PCR reactions, using the Brilliant III Ultra-Fast SYBR^®^ Green and QPCR Master Mix (Agilent Technologies, Santa Clara, CA, USA). The results were presented as the relative expression to the reference gene (GAPDH), assigning the value of 1 to the condition of 0% FBS.

#### 4.2.6. In Vivo Wound-Healing Activity

For the determination of the wound-healing activity (in vivo), the best conjugated dendrimer was selected based on scratch assay. Sprague–Dawley rats (SD) were obtained from the animal house at the Universidad de Talca and were divided into four groups randomly. All animals were sedated with isoflurane and anesthetized with a mixture of ketamine (ketostop, DrangPharmainvetec S.A)/xylazine (Xylaret, Agroland) in a 3:1 ratio (2.2 μL/g weight). Once the animals were anesthetized, they were placed on the surgical plane, the trichotomy and washing of the area were carried out with soap with 0.25% chlorhexidine, and then, a segment of skin was extracted from the area of the back between the scapulae. The surgery was performed with a biopsy punch, followed by the corresponding treatment. Tramadol 20 mg/kg was injected as an analgesic.

The area of the wound was 0.8 cm^2^; this segment was covered by a formulation of GPG3Ca at 0.05 and 0.1%, mixed with a base cream or controls (unsupplemented base cream and saline solution). The treatment was applied once on a daily basis, and photographs were taken every two days for a total period of 16 days. The food and drink intake were also recorded using the same periodicity as well as the behavior and general condition of the animals.

Once the experimental period was over, the animals were sedated by inhalation of isoflurane to subsequently receive a lethal dose of thiopental. Once death was confirmed, a sample of skin and serum was extracted for the respective histological (hematoxylin-eosin and Masson’s trichrome stain) and biochemical (lactate dehydrogenase) analyses. The Institutional Animals Ethical Committee approved the protocol for animal experimentation.

## Figures and Tables

**Table 1:** Measurement of width at half height for the PAMAM amine dendrimer and the different PAMAM conjugated.

PAMAM G3	GPG3Ca	GPG3Cin
1.6 min	0.6 min	0.78 min

**Table 2:** Percentage of cell viability of PAMAM G3 dendrimer and derivatives at 2, 5, and 10 μg/mL.

	PAMAM G3	GPG3Cin	GPG3Ca	Cinnamic Acid	Caffeic Acid	10% FBS
2 μg/mL	94 ± 3%	95 ± 2%	96 ± 2%	97 ± 3%	96 ± 3%	97 ± 2%
5 μg/mL	95 ± 2%	95 ± 4%	96 ± 3%	96 ± 3%	95 ± 4%	97 ± 3%
10 μg/mL	93 ± 3%	93 ± 2%	94 ± 3%	96 ± 2%	96 ± 3%	96 ± 2%

## Data Availability

The data presented in this study are openly available in article.
